# Mild head trauma in elderly patients: experience of an emergency department

**DOI:** 10.1016/j.heliyon.2020.e04226

**Published:** 2020-07-07

**Authors:** Gabriele Savioli, Iride Francesca Ceresa, Luca Ciceri, Fabio Sciutti, Mirko Belliato, Giorgio Antonio Iotti, Sabino Luzzi, Mattia Del Maestro, Gianluca Mezzini, Elvis Lafe, Anna Simoncelli, Giovanni Ricevuti, Federica Manzoni, Maria Antonietta Bressan

**Affiliations:** a Emergency Department, Fondazione IRCCS Policlinico San Matteo, Pavia, Italy; b Intensive Care Unit, Fondazione IRCCS Policlinico San Matteo, Pavia, Italy; c Neurosurgery Unit, Department of Surgical Sciences, Fondazione IRCCS Policlinico San Matteo, Pavia, Italy; d Neurosurgery Unit, Department of Clinical-Surgical, Diagnostic and Pediatric Sciences, University of Pavia, Pavia, Italy; e Neuro Radiodiagnostic, Fondazione IRCCS Policlinico San Matteo, Pavia, Italy; f Geriatric Unit, Università degli Studi di Pavia, Italy; g PhD School in Experimental Medicine, Department of Clinical-Surgical, Diagnostic and Pediatric Sciences, University of Pavia, Pavia, Italy; h Clinical Epidemiology and Biometry Unit, Fondazione IRCCS Policlinico San Matteo, Pavia, Italy

**Keywords:** Neurosurgery, Trauma, Emergency medicine, Internal medicine, Clinical research, Brain injury management, Elderly patients, Hemorrhage, Mild traumatic brain injury, Posttraumatic intracranial hemorrhage

## Abstract

**Introduction:**

We evaluated the risk profile of elderly patients who came to the emergency department for mild head trauma. The primary goal was to determine the difference in the incidence of posttraumatic intracranial hemorrhage (ICH) after minor head injury (MHI). The secondary objective was to assess worse outcome, such as: hospitalization rate, rate of re-admission, need of neurosurgery. We also assess the admission process times and length of hospital stay. The ultimate goal was to optimize the diagnostic-observational management of minor head trauma in elderly patients.

**Material and methods:**

We evaluated all patients with MHI who came to our emergency department during 2017 and 2018. All patients underwent computed tomography.

**Results:**

We enrolled 2325 patients, of whom 1094 were 75 years of age or older. The population was divided into two categories according to age: The “elderly population” was 75 or older, and the younger patients were younger than 75. The elderly population, in comparison with the younger patients, had a higher rate of ICH (12.1% versus 5.1%), a higher hospitalization rate (11.7% versus 5.5%), and a higher rate of readmission within 30 days (6.8% versus 3.2%). The elderly population also had longer admission process times (8 h, 25 min, versus 4 h, 09 min) and longer lengths of hospital stay (9 h, 41 min, versus 5 h, 29 min). Of the younger patients, 92% (versus 41% of the elderly population) did not take any drugs, 6% (versus 39%) were receiving antiplatelet therapy, 1% (versus 13%) took vitamin K antagonists, and 1% (versus 7%) took oral direct-acting anticoagulants. Logistic regression models revealed that a 1-year increase in age raised the risk of bleeding by 2% on average; this finding was statistically significant (odds ratio [OR], 1023/year, *p* < 0.001). The rate of ICH increased significantly after the age of 75, by 180% (OR, 2.82; *p* < 0.001).

**Conclusions:**

These data suggest that age is an independent risk factor for ICH, whereby the age of 75 entails a 180% increase in the risk of bleeding.

## 1 Introduction

Traumatic brain injury (TBI) is one of the most challenging public health problems, worldwide.

Minor head injury (MHI) is one of the commonest reasons for presenting to emergency departments in Italy and abroad. The most of previous epidemiological studies had reported that approximately 75% of the TBI patients could be categorized as mild TBI, according to Glasgow comma scale (GCS) score [1, 2, 3].

Despite medical and pharmacological advances, more effective interventions are needed to improve TBI outcomes, especially in elderly patients [4, 5]. There are discrepancies regarding epidemiology of TBI in the world [4]. These discrepancies could be explained mainly by cultural context of different societies and the age's and gender's distribution [4, 6, 7]. Studies report a different distribution regarding the causes of mild head trauma across different world regions, which are not recognizable through such a holistic statement [6, 8, 9]. Some of these had reported that fall is the most common cause of TBI. Study have often paid less attention or even ignored mild TBI and elder patients [10, 11]. Additionally, older adults with TBI are at greater risk of morbidity and mortality compared to the younger patients [12, 13].

Older people who go to an Emergency Room due to mild head trauma frequently have scalp injuries, headaches (not only in the area of injury), dizziness, nausea with or without vomiting, a temporary loss of consciousness and a short-term memory disorder; epileptic seizures less frequently.

In the elderly, a head injury is almost always the result of an accidental fall, in most cases due to stumbles or inappropriate footwear [14].

Syncope, acute vertigo, episodes of hypotension, non-compensated diabetes mellitus are equally common causes of falls. Almost all elderly patients with mild cranial contusion suffer from one or more internal or neurological diseases such as hypertension, coronary heart disease, chronic heart failure, diabetes mellitus, osteoporosis, M. Parkinson's or dementia. Exact comorbidity frequency data is not available [14, 15, 16].

Elderly patients account for a sizable proportion of these cases. Despite of less lethal outcome of mild TBI, its incidence rate is very high. Additionally, due to the high incidence of TBI in patients who referred to the emergency departments (ED), this higher number of patients lead to a higher work load in the ED, increase health system expenditures, and, even with a lower risk of lethal outcomes, can result in a considerable number of patients with long time disability [17, 18]. The very definition of old age has also been called into question and in recent years task forces of geriatric and gerontological societies (also in Italy) have proposed as old age an age over 75 years [19, 20, 21, 22, 23].

In comparison with younger patients, elderly patients have an higher risk of developing an intracranial hemorrhage (ICH) after head trauma, and their long-term outcome after ICH is worse [24]. As in other neurovascular diseases of surgical interest, older age also represents a risk factor for hemorrhage [25, 26, 27, 28, 29, 30]. Elderly patients are at higher risk than younger patients for complications related to hospitalization and long stays in the emergency department. Our primary objective was to evaluate the difference in the incidence of posttraumatic intracranial bleeding after MHI in elderly and nonelderly patients. Secondary objectives were to assess worse outcome, such as: hospitalization rate, rate of re-admission, need of neurosurgery. We also assess the admission process times and length of hospital stay.

## 2 Materials and methods

### 2.1 Study design

**Figure 1:**
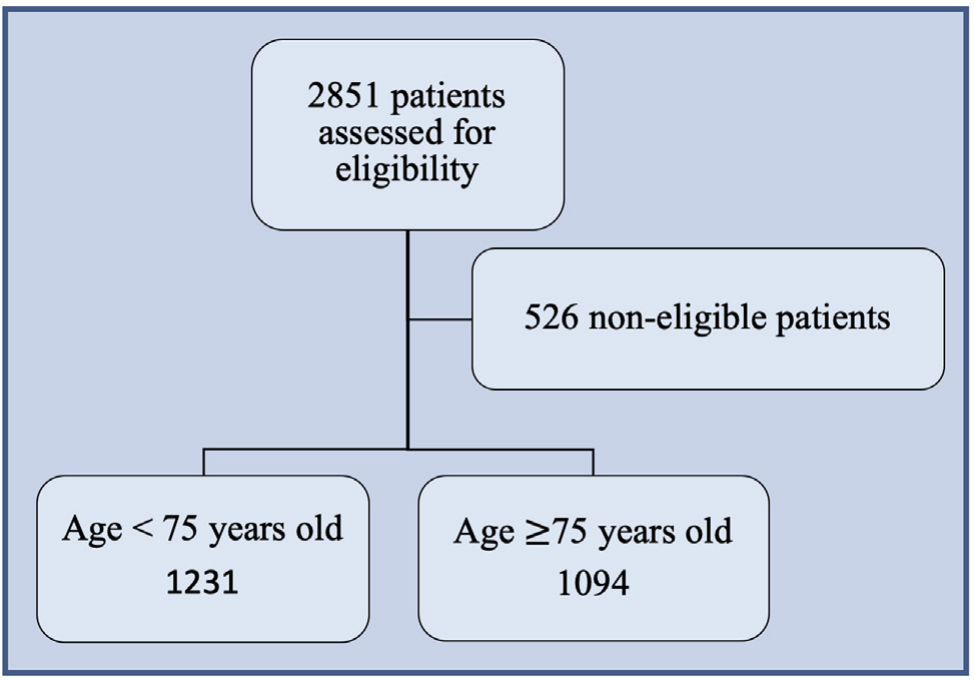
Criteria for the recruiting of patients.

We conducted a retrospective and monocentric observational study of all patients who came to the emergency department of the Fondazione IRCCS Policlinico San Matteo of Pavia, Italy, during 2017 and 2018 for mild head trauma. The population of the study was divided into two categories according to age: Patients in the “elderly population” were 75 or older, and the younger patients were younger than 75. Other risk factors, such as antiplatelet or anticoagulant therapy, were evaluated as well. The primary endpoint was the diagnosis of posttraumatic ICH, both during observation in the emergency department and over the next 30 days. Late bleeding was defined as the diagnosis of ICH when a patient returned the emergency department within 30 days. The study protocol was approved by the local ethical board (Area Vasta Pavia Ethical Committee) and was drawn up in accordance with the statement of the STrengthening the Reporting of OBservational studies in Epidemiology (STROBE) for observational studies (Figure 1).

### 2.2 Inclusion and exclusion criteria

All the patients with mild head trauma were 18 years of age and older, were 18 years of age and older, and had a Glasgow Coma Scale (GCS) score higher than 13 were included. We excluded patients with clinical suspicion of depressed skull fractures or clinical signs of base skull fractures, patients with ICH who had no history of head trauma, and patients with face or neck trauma that did not affect the skull.

### 2.3 Study population

Eligible patients were identified in the electronic database through discharge diagnosis codes corresponding to “cranial trauma,” “ICH,” and “skull/face/neck trauma.” The personal and clinical data of each patient were extracted through our PIESSE digital platform and examined individually to determine whether they could be included in the study. The information had been collected and reported by the emergency department staff and physicians and by medical specialists and included the content of nursing diaries and the results and reports of laboratory and radiological examinations.

For every patient, we also documented demographic data (gender and age), causes and dynamics of trauma, waiting times, admission process times, length of stay in the emergency department, blood pressure, way of arrival (e.g., ambulance), entry code, exit code, hematochemical examinations (particularly hemochrome and clotting examinations), need for hospitalization or surgery, death, and possible return within 30 days after the trauma. All the medical records were viewed and evaluated, and all computed tomographic scans were thoroughly reviewed. All the collected data were stored on a Microsoft Excel spreadsheet and later used for statistical analysis.

This study included 2325 patients, of whom 1094 made up the elderly population.

### 2.4 Statistical analysis

The analyses were carried out with appropriate univariate and multivariate models of logistic regression (with age correction) to test the association between therapy and the ICH rate. Continuous variables were calculated as means and standard deviations; qualitative variables were calculated as numbers and percentages. The results were analyzed in terms of odds ratio (OR) with a 95% confidence interval. The two groups were compared for continuous variables with Student's *t* test or with a nonparameter Mann-Whitney test. Associations between qualitative variables were studied with Fisher's exact test. The significance level was set at alpha = 0.05 (statistical significance for *p* value of <0.05), and all tests were two-tailed. The analyses were conducted with Stata software, version 14 (Stata Corporation, College Station, TX, USA).

## 3 Results

In the electronic database, 2851 patients were identified through a search of discharge diagnosis codes corresponding to “cranial trauma,” “ICH,” and “skull/face/neck trauma.” Of these, 526 were excluded because they did not fulfill inclusion criteria; the remaining 2325 patients were enrolled in the study. The mean age of enrolled patients was 64 years (standard deviation, 22.79); 47% were male and 53% were female. Causes of trauma were, in descending order of frequency, falls (54.71%), accidental collisions (14.90%), minor road accidents (11.93%), syncope (8.05%), violence (5.18%), seizures (0.60%), and sports accidents (0.60%); other causes of trauma were sustained by 4.03% of patients.

### 3.1 Elderly population (≥75 years old)

**Figure 2:**
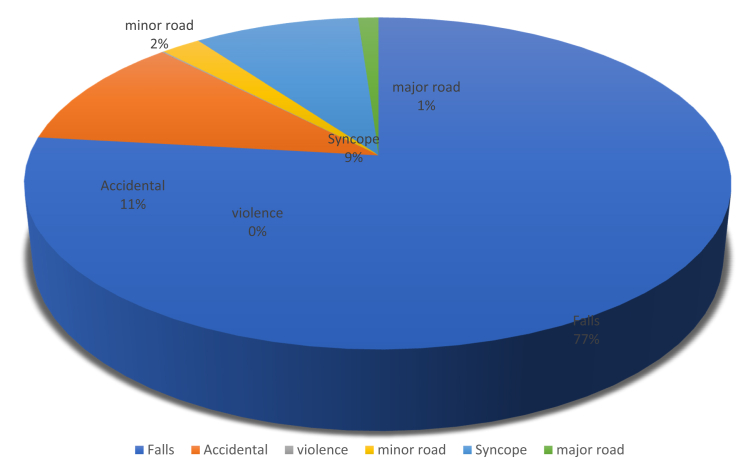
Causes of MHI in people >75 Years.

This population consisted of 1094 patients. Of this population, 17%–18% had total access to health care, and 44% of the population had MHI; 36% were male and 64% were female. Mean heart rate was 78 bpm, mean blood pressure was 145/77 mm Hg, and mean oxygen saturation was 96%. Of the patients, 3% had a GCS score of 14, and 97% had a GCS score of 15. Only 41% did not take any drugs; 39% were receiving antiplatelet therapy, 13% were taking vitamin K antagonists, and 7% were taking oral direct-acting anticoagulants The causes of trauma in this population were, in descending order of frequency, falls (about the 77%), accidental collisions (about the 11%), syncope (about the 9%), minor road accidents (about the 2%), other major road accidents (about the 1%), and violence (about the 0%, more specifically 0.2%)(Figure 2).

At entry to the emergency department, 59% of patients were assigned a yellow code, 40% were assigned green codes, and 1% were assigned red codes. At discharge, however, 78% of patients had been assigned green codes, 17% had been given yellow codes, and 1% still had red codes. They had an average waiting time of 75 min, an admission process time of 505 min, and a length of hospital stay of 581 min.

### 3.2 Young population (<75 years old)

**Figure 3:**
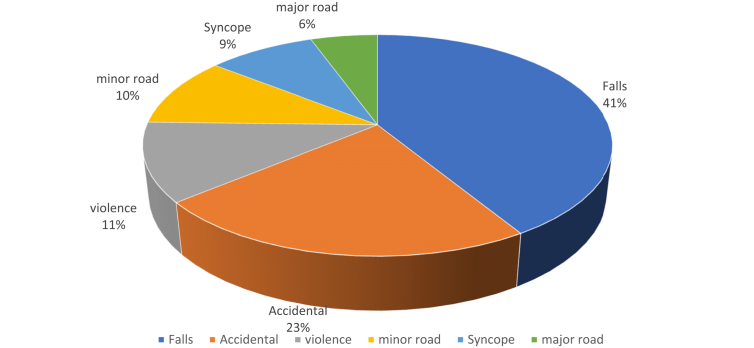
Causes of MHI in people <75 Years.

This population consisted of 1231 patients. Their average age was 43 years, and 40% were female. The mean heart rate was 81 bpm, mean blood pressure was 132/79 mm Hg, and mean oxygen saturation was 98%. One percent had a GCS score of 14 at hospital entry; the rest had GCS scores of 15. Most (92%) did not take any drugs; 6% were receiving antiplatelet therapy, 1% were taking vitamin K antagonists, and 1% were taking oral direct-acting anticoagulants. The causes of trauma in this population were, in descending order of frequency, falls (28%) accidental collisions (18%), violence (10%), minor road accidents (9%), syncope (6%), and major road accidents (4%). Trauma was considered major in 7.2% of cases (Figure 3).

At entry to the emergency department, yellow codes were assigned to 26.4% of the patients, and green codes were assigned to 68%. At discharge, 78.6% had been given green codes, and only 7.9% had yellow codes. The average waiting time was 84 min, the average admission process time was 244 min, and the average length of hospital stay was 329 min.

### 3.3 Outcomes: hemorrhagic complications, hospitalization rates, readmission rates

Older people show worse outcomes about hemorrhagic complications, readmissions, hospitalization rates. In fact, rates of ICH were higher among the elderly patients than among the younger patients (12.1% vs 5.1%), as were hospitalization rates (11.7% versus 5.5%; p < 0.05) and rates of readmission within 30 days (6.8% versus 3.2%, p < 0.05). They also had longer admission processes (8 h versus 4 h) and lengths of emergency room stay (10 h versus 5 h) (Table 1).

**Table 1:** Inclusion and exclusion criteria.

Inclusion Criteria	Exclusion Criteria
Age ≥18 years old	Age <18 years old
GCS ≥13	GCS <13
Minor head trauma	No head trauma
Face/neck trauma affecting the skull	Suspicion or signs of fracture of vault or skull base

Logistic regression models revealed that a 1-year increase in age raised the risk of bleeding by 2% on average; this finding was statistically significant (odds ratio [OR], 1023/year, p < 0.001). The rate of ICH increased significantly after the age of 75, by 180% (OR, 2.82; p < 0.001).

## 4 Discussion

The results of our study show that older people have worse outcomes. This is demonstrated by the highest hospitalization rate, the highest frequency of ICH, the highest rate of readmissions. they need more process time and length of emergency room stay. The relative frequencies of the main causes of mild head trauma were consistent with those reported previously [31]. Major trauma had occurred in 4.7% of our patients, a proportion similar to those found in the main trauma centers of Lombardy. As expected, this type of trauma was more common in the population younger than 75.

The two populations under consideration do not differ in code of priority to the medical examination. In fact, patients who arrive at our ED are first subjected to triage where specialized nurses with basic and advanced business training collect information related to the patient's general data, the main presenting symptoms, and a short history. They then proceed to the measurement of vital signs and conduct a visual inspection. At this stage, based on written protocols (“triage grids”) drawn up mainly based on the evolution of the main symptoms, the patient's medical history, and vital signs, the patients are assigned a priority code for the medical examination and are directed to an area of appropriate intensity of care.

There are 5 levels of priority code for the medical examination in our ED:

a) Red code: immediate entry into the shock room (high-intensity area). It is assigned to patients with severe impairment of vital signs or consciousness.
b) Yellow code with medium care intensity: immediate, or at least within 40 min, entry to the average intensity care area.
c) Yellow code, low care intensity: immediate entry, or at least within 40 min, to the low intensity care area.
d) Green code: assigned to deferred urgency or minor emergencies with a wait of a few hours and entry to the low intensity of care area.
e) White code: non-urgent cases with a wait of a few hours and entry to the low intensity of care area. in both populations there is a clear prevalence of green codes.

Gender data show a slight predominance of women in the general population (53%), but we found a clear difference between the cohorts of patients younger than 75 and those 75 or older, in which the proportions of female patients were 40% and 64%, respectively. This is attributable to the longer life expectancy of women than of men; thus, women account for a greater proportion of the older population of Italy. In addition, young men are more likely to suffer trauma because of work, behavior, and a higher rate of drug and alcohol intake. This is probably also the reason why the rate of ICH was 40% higher among the men than among the women in our study.

As with investigations of other neurosurgical diseases, many recent studies of ICH have focused on improving the overall management. In recent years, the average age of the population has steadily increased, and at the same time, the mental and physical performance of men and women living in economically developed countries has improved to such an extent that the Italian Society of Gerontology and Geriatrics has postulated a new definition of elderly, moving the threshold from 65 years to 75 years.

As presumably, older people take more therapy than younger patients, in particular more anticoagulant and anti-aggregator drugs. We then carried out an analysis to see if the worst outcomes found in older people were not actually simply due to a higher prevalence of this type of therapies.

In this study, the median age of patients with hemorrhage (79 years) was greater than that of patients without bleeding (75 years; *p* < 0.0001) Appropriate logistic regression models revealed that a 1-year increase in age raises the risk of bleeding by 2% on average in a statistically significant way (OR, 1023/year; *p* < 0.001).

We noted a major increase in the rate of ICH after the age of 75. The age of 75 entailed a 180% increase in the risk of bleeding (OR, 2.82; *p* < 0.001) in comparison with younger ages, when the analysis was controlled by gender. Age was an independent risk factor for bleeding. Male gender was associated with an increase of about 42% in the rate of ICH (OR, 1.43; *p* = 0.03) (Table 2).

**Table 2:** Logistic regression model to test the association between hemorrhage risk and age category - 75 Years.

	Odds Ratio	Std. Err.	z	P>|z|	[95% Conf. Interval]	
Age_75	2.570	.452	5.39	0.000	1.826	3.634

We believe that the age of 75 should be considered an independent risk factor in the management of MHI and that patients aged 75 or older who suffer MHI should undergo brain computed tomography. In selecting the medical or surgical treatment to be performed, clinicians should also evaluate minimally invasive options for such patients [32, 33]. The STROBE guidelines (except for Canadians) suggest that in patients aged 65 and older, such extra care is necessary only in the presence of other risk factors (loss of consciousness, amnesia, or concurrent therapy with anticoagulants).

The study also highlights that the elderly population is at risk of worse outcomes such as a greater need for hospitalization and hospitalization and more frequent readmissions to the emergency room at 30 days. That the elderly population is more at risk of worse trauma outcomes is also known for other trauma categories, but the study points out that this also applies to mild head trauma.

With regard to the need for an operating room, the elderly group has a higher frequency of need for surgery. In our study, however, the figure, perhaps also due to the small number of people undergoing surgery, does not reach statistical significance.

## 5 Conclusions

Our study focuses on the risk of worse outcomes in older people also in light of the new proposed of old age. The data show that the age is an important independent risk factor in patients with mild head trauma. In particular age over 75 is a risk factor for worse outcomes such as intracranial bleeding, hospitalization and readmission. Although the group of the elderly more often needs the operating room the figure does not reach statistical significance. Because of this, we consider it useful to have a careful evaluation of these patients in ER and to consider for them an appropriate observation period and a CT scan.

## Declarations

### Author contribution statement

G. Savioli: Conceived and designed the experiments; Performed the experiments; Analyzed and interpreted the data; Contributed reagents, materials, analysis tools or data; Wrote the paper.

I. Ceresa, L. Ciceri, F. Sciutti, M. Belliato, G. Iotti, S. Luzzi, M. Del Maestro, G. Mezzini, E. Lafe, A. Simoncelli, G. Ricevuti and B. Antonietta: Performed the experiments; Contributed reagents, materials, analysis tools or data.

F. Manzoni: Performed the experiments; Analyzed and interpreted the data; Contributed reagents, materials, analysis tools or data.

### Funding statement

This research did not receive any specific grant from funding agencies in the public, commercial, or not-for-profit sectors.

### Competing interest statement

The authors declare no conflict of interest.

### Additional information

No additional information is available for this paper.
